# Ultra-Processed Food: The Tragedy of the Biological Commons

**DOI:** 10.34172/ijhpm.2022.7557

**Published:** 2022-12-26

**Authors:** Norah Campbell, Sarah Browne, Marius Claudy, Kathryn Reilly, Francis M. Finucane

**Affiliations:** ^1^Trinity Business School, Trinity College Dublin, Dublin 2, Ireland.; ^2^School of Business, University College Dublin, Dublin, Ireland.; ^3^Irish Heart Foundation, Dublin, Ireland.; ^4^School of Medicine, CMNHS, University of Galway, Galway, Ireland.; ^5^Saolta University Health Care Group, Galway University Hospital, Galway, Ireland.

## Reformulation: A Major Public Health Strategy

 An important contributor to the rising global prevalence of obesity^1^ has been the widespread availability of cheap, unhealthy ultra-processed foods (UPFs). These are “formulations of ingredients, mostly of exclusive industrial use, typically created by a series of industrial techniques and processes.”^2^ UPFs are nutritionally inferior to unprocessed, minimally processed or processed foods, containing significantly higher proportions of free sugars, total and saturated fats, salt and energy density, and lower proportions of protein, fibre, vitamins and minerals.^3^ They are affordable, hyper-palatable, stimulate repeat purchase, are aggressively marketed and branded, and highly profitable.^4^ The evidence is now unequivocal that UPFs are a significant driver of obesity,^5^ diabetes,^6^ increased morbidity and mortality^7^. UPFs now comprise over half of all food intake in America^8^ and Britain,^9^ radically displacing healthier, less processed food from the diet.^10^

 Reformulation – the reduction of saturated fats, sugar and salt in food – has long been considered a strategy to mitigate the public health risks associated with unhealthy dietary patterns globally.^11^ Food reformulation is perceived as a ‘win-win’^12^ because unlike other public health nutrition policies it has the potential to also benefit the UPF industry, as the focus is on changing the nutrient profile of a product rather than decreasing its overall consumption.^13^ However, public health experts have argued that there are no such things as ‘healthy’ UPFs: replacing sugars with artificial sweeteners, for example, is not a strategy that leads to a greater share of unprocessed food in the diet.^11,13^ In order to assess the policy of reformulation, it is necessary to consider the system dynamics between the scale, scope and speed of food industry *reformulation* efforts against the scale, scope and speed of their *formulation* efforts. In other words, reformulation should be a ratio which measures UPF innovation (the creation of new products, product line extensions, delivery channels, snacking occasions and portion sizes) against the reduction of sugar, saturated fats and salt in this type of food.

 An emblematic case in action is Ireland. The Irish government, as part of its national obesity strategy, has recently sought to develop a “reformulation roadmap” in a voluntary partnership with the food industry. Nutritional epidemiological data on UPF consumption patterns in Ireland are sparse. Public health actors have had to rely on data presented in food industry reports that describe notional improvements in population dietary patterns due to industry-led reformulation. These reports are prone to sample selectivity and bias.^14^ However, commercial databases such as Euromonitor^15^ now offer data on the volume, sales, nutrient composition, intake, and brand share for UPFs which could serve as a proxy for measuring changes in patterns of population dietary behaviour over time. Such databases are recognised as providing valid and important insights for public health nutrition researchers.^16^

## Ultra-Processed Food Sales in Ireland

 Commercially-available databases such as Euromonitor Nutrition^15^ now give data on the volume, sales, nutrient composition, intake, and brand share for UPFs across 54 countries, collated from primary and secondary data sources, including store audits, interviews with companies, sales data, nutrient tracking, and company reports. The advantage of such databases is that they are predominately used by food industry actors to gain market intelligence and competitive advantage, offering a more unfiltered picture of the industry. We downloaded data relating to food purchases and nutritional content in Ireland from the Euromonitor service^15^ through our institutional subscription. Specifically, we obtained data relating to the total weight of snack foods purchased in Ireland each year between 2007 and 2020, as shown in Figure. These per capita purchases are estimated on the basis of total retail volume, which excludes sales from fast food outlets, deli counters, meat snacks, dairy, unpackaged pastries, baked goods, breakfast cereals, sweet spreads, hot drinks or weaning foods, energy drinks and alcohol products. Thus, these data are likely to represent a significant underestimate of overall UPF purchasing in Ireland.

**Figure:**
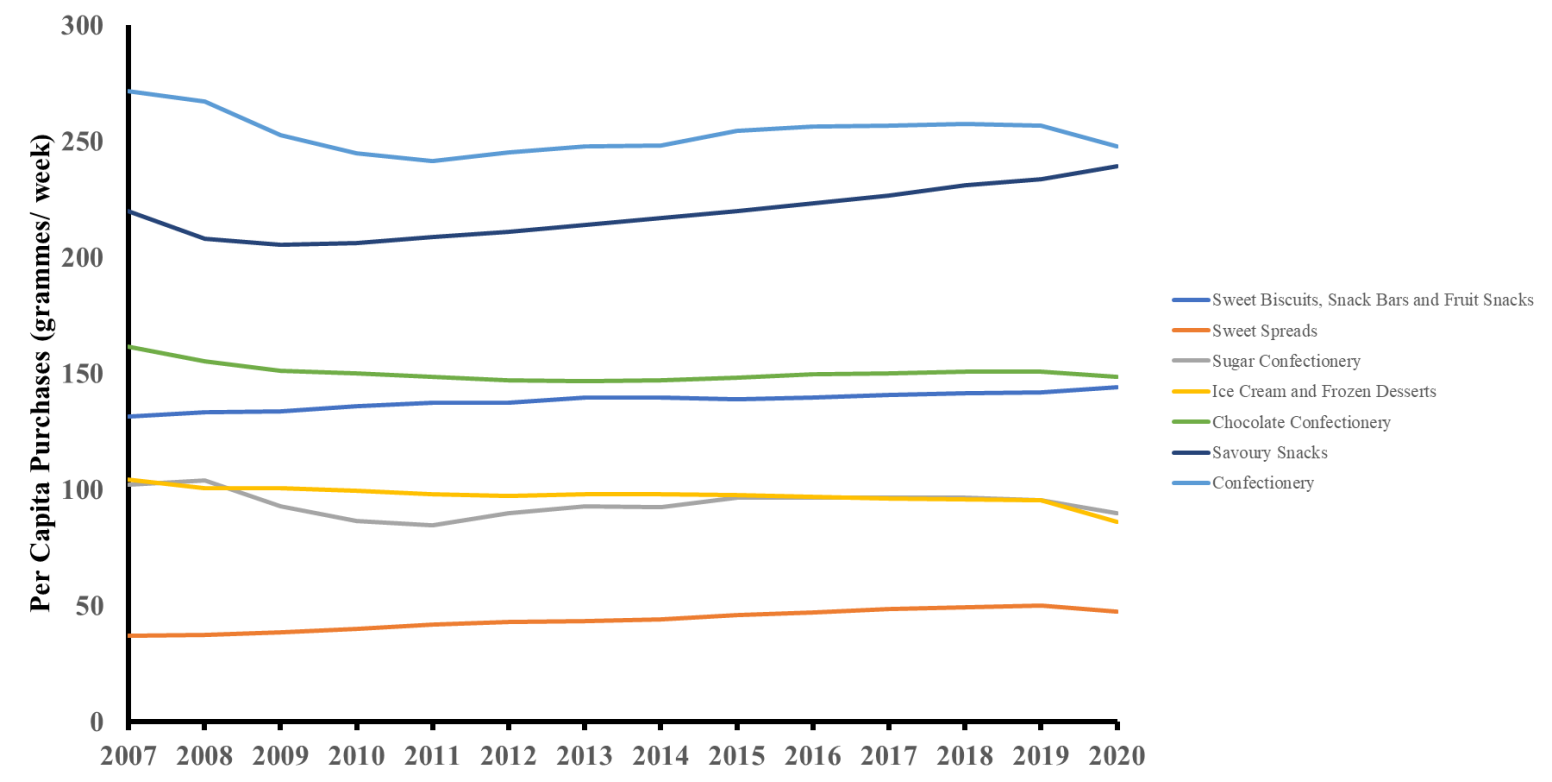
Weekly Per Capita Purchasing (in Grammes) of Various Types of Ultra-Processed Foods in Ireland Between 2007 and 2020, According to Euromonitor.

 The data suggest that in the last 15 years snack food purchases have remained static, with small reductions in confectionery, chocolate confectionery, sugar confectionery, ice cream and frozen desserts, but also small rises in per capita purchases of savoury snacks, sweet biscuits, snack bars and fruit snacks and sweet spreads. In effect, purchases of these products have “stabilised” at an extremely high level, creating a new normal. For example, although confectionery sales declined by nine percent (from an average per person per week of 272 grammes in 2007 to 248 grammes in 2020) – the largest reduction in any snack domain – current consumption is still the equivalent of more than five packets of “Skittles^®^” (weighing 45 grammes) per week for every man, woman, and child in Ireland. Weekly chocolate confectionery purchasing declined by nine percent, from 162 to 149 grammes per week. That is still equivalent to three standard Mars^®^ bars per week. Savoury snack purchasing increased by nine percent from 220 to 239 grammes per week, equivalent to a packet of crisps every day. There was also 9% increase in the biscuit category, from 132 to 144 grammes per week, equivalent to ten digestive biscuits. There was a 17% reduction in ice cream purchasing, from 104 to 86 grammes per week, equivalent to a Magnum^®^ bar each week. The cumulative burden of UPF consumption on population dietary health (and non-communicable disease risk) is likely to be very large, even if this is not apparent when considering individual categories or individual brands.

## Leanwishing – the Logic of the Ultra-Processed Food Industry

 There is a growing awareness among academics and researchers of problematic corporate marketing practices, such as greenwashing, pinkwashing, wokewashing, and leanwashing.^1,17,18^ ‘X-washing’ occurs where a veneer of corporate social responsibility is deployed to intentionally mask business-as-usual operations, and deflect stakeholder attention towards visible but ultimately superficial changes in corporate agendas. Specific to the food industry, the critical management theorist Aneel Karnani and colleagues define leanwashing as “the relations and marketing activities of a firm that deceptively promote the perception that the firm is helping to solve the obesity problem and that deflect attention from the fact that it is directly contributing to the obesity crisis.”^18^ In other words, leanwashing, like all other forms of ‘X-washing’ involves the intent to cynically deceive, manipulate, and aggressively persuade stakeholders.

 However, the reformulation efforts of the UPF industry can be accompanied with the belief on its part that its efforts to tackle the obesity crisis are genuine, commensurate with the scale of the problem and well-intentioned. To mark this more nuanced corporate logic we introduce the term “*leanwishing ”*– the use of corporate responsibility practices by the food industry with the sincere hope that these will reverse the obesity crisis which now impacts most countries around the world, and the trends of which no country has managed to reverse using existing policy mechanisms. In other words, leanwishing is investing in the reformulation of snack foods while simultaneously disavowing the systemic dynamics of *formulation*. “Formulation” comprises all food innovation that stimulates a desire for energy dense, nutritionally empty foods: new product development, new flavours, editions, package sizes, snacking occasions and channels, as well as the infiltration of previously healthful food categories with UPF variations. Reformulation is, we argue, at a pace and scale that is not commensurate with formulation in this industry.

## A Tragedy of the Biological Commons?

 We have not met a single snack food executive who wished for their consumers to be overweight. Rather, those managing snack brands face a double-bind: a fiduciary responsibility to grow the market share, and a social responsibility to decrease consumption of unhealthy products. Applying a critical marketing and behavioural economic frame to this public health challenge yields what we consider to be a “tragedy of the biological commons.” In one of the most cited papers in history, the population ecologist Garrett Hardin asked the reader to “picture a pasture open to all.”^19^ Each herder will try and put as many cattle as possible on this commons, because they get a direct benefit from their own animals grazing, and suffer only a postponed cost from when their own and others’ cattle overgraze and the commons deteriorates. In other words, each herder will be motivated to add more and more animals because they get a direct advantage from their own animals, but shoulder only a small and delayed proportion of the costs that arise from overgrazing. Hardin sums up the situation of the commons: “Therein is the tragedy. Each man is locked into a system that compels him to increase his herd without limit – in a world that is limited. Ruin is the destination toward which all men rush, each pursuing his own best interest in a society that believes in the freedom of the commons.”

 Hardin’s paper is a foundational text used in many disciplines to describe how, under conditions of any commons, action that is individual and rational can produce collectively catastrophic outcomes, such as overpopulation, climate change or nuclear armament. We argue that population health is another limited and shared resource that mirrors the tragedy of the commons. The herders are UPF-producers, and unhealthy food products are structurally analogous to a herd of animals, competing for a shared but limited common pasture on which to graze. The population’s body is that common pasture, being open to all companies which compete intensely for a share of it, but limited because there is only a certain amount of food that can be healthily consumed. This way of framing the problem of obesity is not focussed on overconsumption, but rather overproduction. If public health actors can consider the tragedy of the biological commons, where population dietary health is an open and limited resource, we might better comprehend the inevitable logic of the snack food industry, and how individually rational industry actors collectively overgraze the commons, however unintentionally.

 Certain characteristics of Hardin’s original commons pertain directly to the biological commons. First, as Hardin notes, a commons can be sustainable in situations of low population density. This is equivalent to low levels of snack production and availability until the 1980s. Since then, Western societies and lower income countries have experienced a steep rise in the production of UPFs, driven by innovation in food science, hyper-competitive industry dynamics, and innovations in consumer profiling and targeting.^3^ But despite the resultant crisis, UPF companies market products as if there were no other herders on the commons. Take for example an opinion piece in the *London Times* newspaper in 2021, where the managing director of Hariboâ (a sweet manufacturer) implored the public to see sense in the face of regulation of snack food manufacturers to the biological the commons: “Haribo’s goal is to create moments of childlike happiness. We believe an occasional sweet treat with loved ones is a key part of happiness. Given the year we’ve had, small moments of happiness are sorely needed.” This emblematises the entire logic of the snack food industry – creating moments, “treatwise^®^,”^20^ small indulgence, special celebration, reward yourself, mindful snacking, a balanced diet. Viewed individually, each company acts rationally, aiming to persuade consumers to indulge only in an occasional treat. Viewed cumulatively, that is, a logic that applies to thousands of competitors, it is a limitless production on a commons that is limited.

 A second aspect of the original commons is that once individuals become aware that others are exploiting the commons, they are more likely to increase their own exploitation of it too. This phenomenon of *stimulated exploitation* has been observed in commons dilemmas as diverse as forestry and traffic congestion. In the food industries, observing the success of competitors’ UPFs in the market place is likely to further spur industry efforts to create hyper-palatable, nutritionally empty products that inadvertently contribute to populations’ worsening dietary patterns.

 Thirdly, Hardin described how an individual exploiting the commons cannot restrain themselves by recourse to their individual conscience. The entire logic of ‘responsibility’ is dangerously ineffective because it produces feelings of guilt and anxiety in non-co-operators, while doing nothing to reduce the exploitation of the commons. The inauguration and spread of corporate social responsibility only induces guilt and anxiety in corporate actors, attempting, in Hardin’s words, to “browbeat a free man in a commons into acting against his own interest.”^19^ If the snack food executive radically changed the formulation of UPFs in an attempt to protect and improve public health, they know that others will flood in to fill the void. Instead, what is needed, following Hardin, are “definite social arrangements.” There is a difference between slowing down the rising prevalence of overweight and obesity and reversing it. Taxation, advertising legislation, channel restriction, plain packaging, and mandatory reformulation would support the latter. Undertaken together, such measures could partially enclose and protect the biological the commons. Arguably, it could also significantly reduce the anxiety of industry actors who are currently obliged to engage in two contradictory actions: to grow overall consumption while at the same time reformulating (some of) their products.

 For policy-makers, knowing that the biological commons is a limited resource with the attendant characteristics of other forms of commons might lead to a new win-win solution: Regulation of the biological commons would clarify the roles and remits of the food industry, and would relieve commercial actors of the strain of finding ways to circumvent the vague obligations that currently exist. The voluntary paradigm that has governed food reformulation does not recognise the structure of the problem and disavows the logics of the UPF industry. Ultimately, this means that there is an urgent requirement for statutory regulation of reformulation in the general public interest. Such “definite social arrangements” might come as a welcome relief to the lobbyists, marketers and advertisers who herd on the biological commons with inadequate solutions at their disposal to limit their unsustainable overgrazing.

## Ethical issues

 Not applicable.

## Competing interests

 Authors declare that they have no competing interests.

## Authors’ contributions

 NC drafted the concepts, and reviewed the drafted. SB, MC, and FF investigated the case of ultra-processed food data, and co-wrote the manuscript.
